# Correction: Mathew et al. Metabolic Signature of Warburg Effect in Cancer: An Effective and Obligatory Interplay between Nutrient Transporters and Catabolic/Anabolic Pathways to Promote Tumor Growth. *Cancers* 2024, *16*, 504

**DOI:** 10.3390/cancers16091627

**Published:** 2024-04-24

**Authors:** Marilyn Mathew, Nhi T. Nguyen, Yangzom D. Bhutia, Sathish Sivaprakasam, Vadivel Ganapathy

**Affiliations:** Department of Cell Biology and Biochemistry, Texas Tech University Health Sciences Center, Lubbock, TX 79430, USA; marilyn.mathew@ttuhsc.edu (M.M.); nhi.t.nguyen@ttuhsc.edu (N.T.N.); yangzom.d.bhutia@ttuhsc.edu (Y.D.B.); sathish.sivaprakasam@ttuhsc.edu (S.S.)

In the original publication [1], we noticed an error in Figure 1B. There should have been no involvement of PFK1 regulation by ATP or F2,6-BP. This figure describes anerobic glycolysis in normal cells, and this process is associated with reduced ATP levels due to the lack of mitochondrial oxidative phosphorylation. This relieves the inhibition of PFK1 by ATP, thus promoting glycolysis. The corrected Figure 1 should be as follows.

The authors apologize for any inconvenience caused and state that the scientific conclusions are unaffected. The original publication has also been updated.

## Figures and Tables

**Figure 1:**
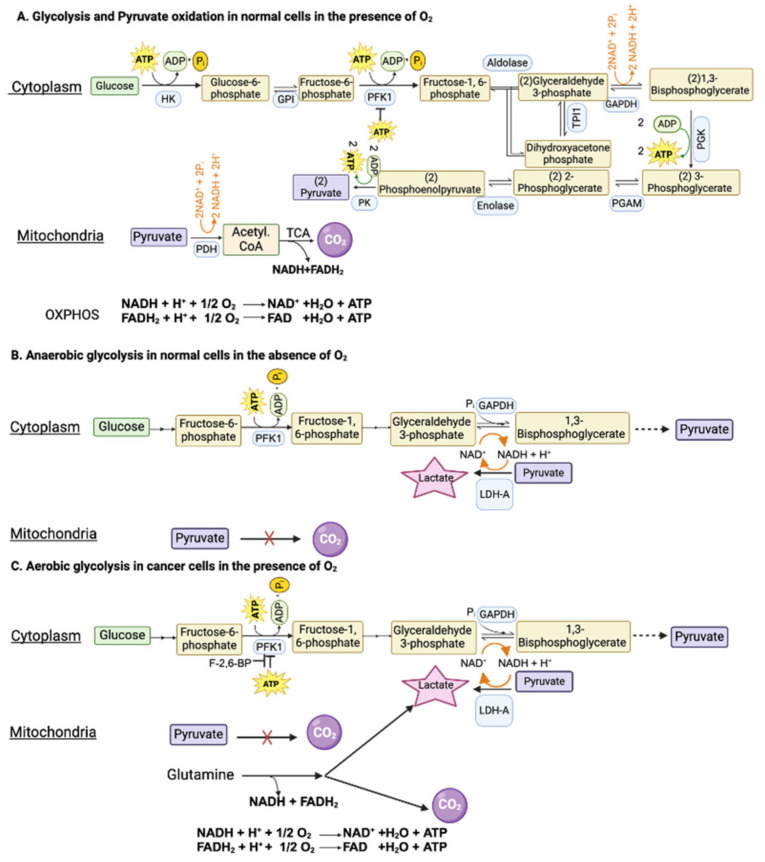
Glycolysis in normal cells and in cancer cells in the presence and absence of oxygen. GAPDH, glyceraldehyde-3-phosphate dehydrogenase; PFK1, phosphofructokinase-1; F-2,6-BP, fructose-2,6-bisphosphate.
